# Evaluating the Drought Tolerance of Seven Potato Varieties on Volcanic Ash Soils in a Medium-Term Trial

**DOI:** 10.3389/fpls.2021.693060

**Published:** 2021-06-25

**Authors:** Ingrid Martínez, Manuel Muñoz, Ivette Acuña, Marco Uribe

**Affiliations:** Instituto de Investigaciones Agropecuarias, INIA Remehue, Osorno, Chile

**Keywords:** drought index, irrigation, tuber size distribution, marketable tuber yield, Andisols, water stress

## Abstract

One of the main factors limiting the productivity of potatoes (*Solanum tuberosum* L.) is water stress. Two irrigation systems: full irrigation (I) and rainfed conditions (R), were compared over the growing seasons from 2012–13 to 2019–20. The evaluated varieties were Desiree, Karú-INIA, Patagonia-INIA, Puyehue-INIA, Yagana-INIA, Yaike, and Porvenir. This study determined (i) the yield and tuber size distribution, (ii) their relationship between productivity and environmental conditions, and (iii) the most drought-tolerant varieties based on drought tolerance indices. Nine indices including yield index (YI), tolerance index (TOL), mean productivity (MP), geometric mean productivity (GMP), harmonic mean (Harm), stress tolerance index (STI), harmonic mean productivity (HMP), yield reduction (Yr), and stress susceptible index (SSI) were calculated by using tuber yield under I and R conditions. Tuber yield under R conditions decreased by 27 and 34%. However, the highest yield under R conditions occurred in years with more precipitation between 60 and 120 days after planting (DAP; ±60 mm). Under R conditions, the varieties Porvenir, Patagonia-INIA, Yaike, and Puyehue-INIA showed more tolerance to water stress. Water stress negatively affected tuber size distribution, reducing the production of tubers with size >65 mm by 50–60%. The best indices to study drought tolerance were TOL, MP, GMP, Harm, STI, and HMP. This study suggests that in southern Chile, an area with big yield potential, typically cultivated as rainfed, with cool temperate climate conditions and favorable soil properties for potatoes, as Andisols, available rainfall is still a constraint for yield. Therefore, using more water stress-tolerant varieties and providing supplementary irrigation between 60 and 120 DAP are critical to optimize yield and avoid the failure of the crop in years with remarkably low precipitations, which will be more pronounced in the future according to weather trends. These results exemplify how much we can lose in productivity in rainfed conditions even in one of the most favorable areas for growing potatoes in the world and how risky this situation can be for the performance of the potato farms in the future.

## Introduction

The potato (*Solanum tuberosum* L.) is the fourth most important cultivar worldwide, with a global annual yield of 370 Mt, with 17.3 million ha under cultivation (FAOSTAT, 2020). One of the main factors limiting the productivity of potato production is the need for more effective water management (Fleisher et al., 2015). Plants differ in their capacity to use water efficiently. The mechanisms responsible for regulating plant water use remain only partially understood. Mbava et al. (2020) identified three important factors that account for differences in water use efficiency (WUE): (i) carbon-fixing or C4 plants make more efficient use of water than do C3 plants, (ii) plants in warm, humid climates make more efficient use of water than plants in cold climates, and (iii) soil texture has a significant effect on water retention, clay soils facilitating better WUE than silty soils. Like C3 plants, potatoes make less efficient water use, because of which their productivity varies significantly owing to differences in climate, irrigation, and soil management, with better yields when the availability of water and light are not limiting factors (Zhao et al., 2012; Mi et al., 2015; Mbava et al., 2020). Several authors have shown that water stress affects physiological mechanisms, which can be genotype-dependent. The most severe effects of water stress on potatoes are differences in stolon, tuber formation, yield, and tuber size distribution (Monneveux et al., 2013; Yuan et al., 2013; Aliche et al., 2019, 2020; Gouveia et al., 2019).

Stress factors like drought and excess UV radiation that negatively affect plant yield will be more common in the coming decades due to climate change (Wang and Frei, 2011; Gregory and Marshall, 2012). Environmental stress because of decreased precipitation and changes in temperature affect potato yields, especially in dry regions (Aliche et al., 2019). Although the productivity of irrigated agricultural systems in semiarid areas has increased, water scarcity and the cost of irrigation limit the expansion of these systems (Mbava et al., 2020). Fang and Xiong (2015) argued that plants can be divided into four categories in terms of drought resistance: drought avoidance, drought tolerance, drought escape, and drought recovery. Of these, drought avoidance and drought tolerance are the main survival mechanisms. Zhang et al. (2018) described drought avoidance as the capacity to increase the root/shoot ratio to improve water absorption and close the stomata to reduce transpiration, while the mechanism of drought tolerance is related to the physiological parameters of osmotic adjustment.

To assess genotype responses to water stress, we used indices based on plant resistance or susceptibility. Based on mathematic relationships, selection parameters were used to identify genotypes with high yield potentials under favorable or stressful conditions (Mardeh et al., 2006), as well as genotypes tolerant to high altitudes (Noerwijati and Budiono, 2015), saline soils (Krishnamurthy et al., 2016; Jamshidi and Javanmard, 2018), and contrasting water availability conditions (El-Hendawy et al., 2017; Choudhary et al., 2018). According to Fernandez (1993), genotypes can be divided into four categories based on their associated yields: (1) genotypes with high yields under conditions with or without stress, (2) genotypes with high yields under conditions without stress, (3) genotypes with high yields under stressful conditions, and (4) genotypes with low yields under either condition.

Around 16% of agricultural production in Chile corresponds to potato production (41.268 ha), of which 70% is concentrated in the center-south of this surface area (2018–19, INE). Moreover, a high percentage of this area is dominated by soils derived from volcanic materials, accounting for 50–60%, which is important for agriculture (Valle et al., 2018). Owing to their andic properties, these soils are different from other soils because of their high soil organic carbon and nutrients content, low bulk density, and capacity to store water (Valle et al., 2018; Clunes et al., 2021). Studying the connections between yields, soil and climate shed light on drought resistance mechanisms. The objectives of this study were to assess seven potato varieties (*S. tuberosum* L.) under conditions of irrigation and rainfed in a medium-term trial to determine: (i) the yield and tuber size distribution, (ii) their relationship between productivity and environmental conditions during their development, and (iii) the most water stress-tolerant varieties using drought-tolerant indices in volcanic ash soils.

## Materials and Methods

### Site Description and Field Experiment

Seven potato varieties (*S. tuberosum* L.) were selected to study their water stress tolerance during the 2012–13 to 2019–20 seasons (except for the 2013–14 season) at the Instituto de Investigaciones Agropecuarias (INIA Remehue) in Osorno, Chile (40°35'S, 73°12'W, 72 m.a.s.l.). The selected varieties, except for Desiree (1962, the Netherlands), had been released by INIA: Yagana-INIA (1983), Karú-INIA (2002), Patagonia-INIA (2009), Puyehue-INIA (2011), Yaike (2019), and Porvenir (2019). Due to an institutional decision, the varieties released from 2019 do not have the suffix of INIA, as the case of Yaike and Porvenir. Desiree and Karú-INIA were originally included but were stopped after five seasons (2017–18) to include new genotypes. Porvenir was included in season 2014–15, completing six seasons.

The soil was volcanic in origin, an Andisol of the Osorno series, classified as a Typic Hapludand [Centro de Información de Recursos Naturales (CIREN), 2003], textural class: silty loam, characterized by a high level of phosphorous retention and low bulk density (Valle et al., 2015). To determine soil physical properties, undisturbed soil cores (diameter 5 cm, height 5 cm) were collected in 10 cm increments to 100 cm depth, with three replicates. At the Bromatology Laboratory of INIA Remehue, the cylinders were weighed and dried in an oven at 105°C for 24 h to determine the water content of the soil, based on which the bulk density and porosity were calculated. Colors in the soil profile were identified with the Munsell Soil Color System (Munsell, 2009).

The experiment was carried out with a split-plot randomized design with three replicates; the irrigated (I) condition and rainfed (R) condition were assigned to the main plot and the varieties to the subplots. The size of each subplot was 4.5 m in length by 3 m in width (13.5 m^2^ in total area), and the planting pattern was 0.75 m between rows × 0.33 between plants. In the I condition, drip irrigation was homogenously applied after emergence during the crop cycle. The total quantity of water received by the crop cycle over the seven seasons varied between 450 and 600 mm based on climatic conditions. After 1 week from emergence, all plots in the I condition were irrigated weekly with 37.5–50 mm of water. This amount is considered to be enough to satisfy the crop evapotranspiration estimated during the crop cycle. The R condition (no irrigation applied) was included to represent water-limited conditions for the crop, which considered the rainfall during the growing season. The plots were fertilized with 120 kg of N ha^−1^, 350 kg of P_2_O_5_ ha^−1^, and 160 kg of K_2_O ha^−1^. The duration of the phenological development was between 130 and 150 days. This coincides with the usual crop management in the area, conditioned by the climate requiring potatoes to be harvested before the end of March.

### Climatic, Crop Evaluations, and Drought Tolerance Indices

Climatic measurements were taken every day at the experimental station during the period from seeding to harvest. To adjust the determined crop coefficients for a potato to site-specific climatic conditions, a heating unit-based Growing Degree Days (GDD) (McMaster and Wilhelm, 1997), which was calculated with the equation below:

$$\sum =\left\{\frac{Tmax+Tmin}{2}\right\}-Tbase$$

where Σ is the number of days from emergence to harvest; *Tmax* is the highest temperature; and *Tmin* is the lowest temperature. The monthly accumulated data for the GDD were considered at a *Tbase* of 4°C as the minimum temperature for potato growth (Ahmadvand et al., 2009). Accumulated precipitation, average temperature, and solar radiation at 60 and between 60 and 90, and 90 and 120 days after planting (DAP) were also considered. Weather data (daily maximum and minimum air temperature, precipitation, and solar radiation) were obtained from an automatic weather station at the experimental station at INIA Remehue.

The following yield traits were systematically collected at the harvest date in two internal rows in each subplot, each 4.5 m in length (6.75 m^2^ in total area): yield of marketable tubers (>65 mm size), the yield of seed tubers (25–65 mm size) and non-marketable tubers (<25 mm size). In Chile, the seed tuber size to be marketed is regulated by the SAG (The Agricultural and Livestock Service) certification standard. The assessments were conducted in each experimental unit when 70% of the plant leaves were yellowish in color.

Various drought tolerance indices were calculated to estimate the stability in yield under I and R conditions: yield index (YI), tolerance index (TOL), mean productivity (MP), geometric mean productivity (GMP), harmonic mean (HARM), stress tolerance index (STI), harmonic mean productivity (HMP), yield reduction (Yr), and the stress susceptibility index (SSI). The formulas for these indices are presented in Table 1.

**Table 1:** Description of the drought tolerance indices based on tuber yield.

Drought tolerance indices		Formula	References
YI	Yield index	Y_s_/X_s_	Lin et al., 1986
TOL	Tolerance index	Y_p_ − Y_s_	El-Hendawy et al., 2017
MP	Mean productivity	(Y_s_ + Y_p_)/2	El-Hendawy et al., 2017
GMP	Geometric mean productivity	(Y_p_ × Y_s_)	Krishnamurthy et al., 2016
HARM	Harmonic mean	(2 × Y_s_ × Y_p_)/(Y_p_ + Y_s_)	Nikneshan et al., 2019
STI	Stress tolerance index	(Yp × Ys)/X_p_^2^	Fernandez, 1993
HMP	Harmonic mean productivity	(2 × Y_p_ × Y_s_)/(Yp + Ys)	Fernandez, 1993
Yr	Yield reduction	1 − (Y_s_/Y_p_)	Golestani and Assad, 1998
SSI	Stress susceptibility index	[1 − (Y_s_/Y_p_)]/[1 − (X_p_/X_s_)]	Nikneshan et al., 2019

### Statistical Analysis

An ANOVA with a split-plot randomized into a three-block design was used to assess the effects of treatments on crop productivity. Means were tested using the Tukey method at a significance level of *p* < 0.05. Regression analyses were performed to assess the associations between variables. Relationships between analyzed variables were established through principal components analysis (PCA) and correlation coefficients (*p* > 0.05) using the Infostat statistical analysis software (Di Rienzo et al., 2009).

## Results

### Environmental Conditions and Tuber Yield

**Figure 1:**
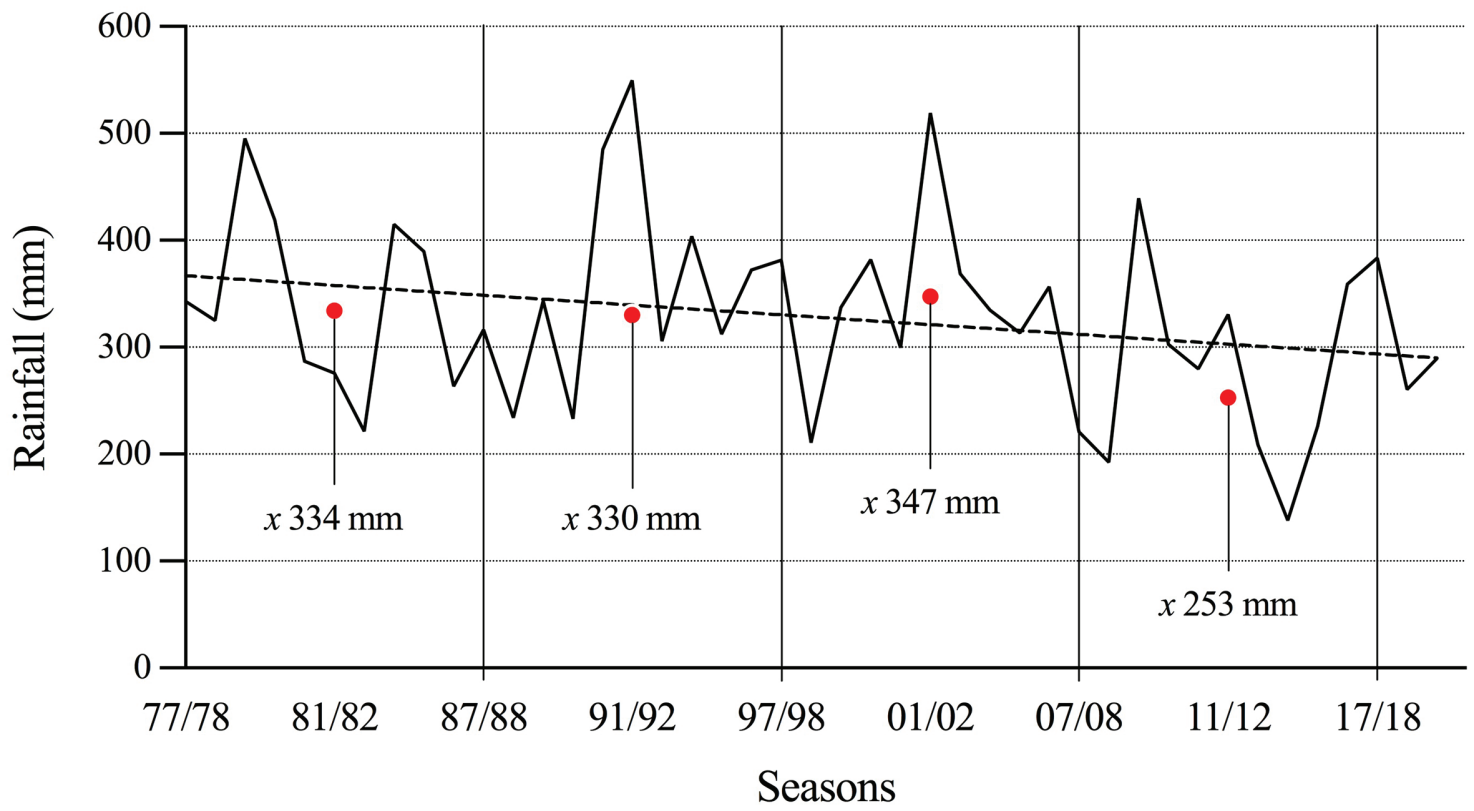
Long-term accumulated rainfall amount during the potato growing season from 1977/78 to 2019/20 in the study area. Each red point represents de average rainfall amount during the last 10 years.

**Figure 2:**
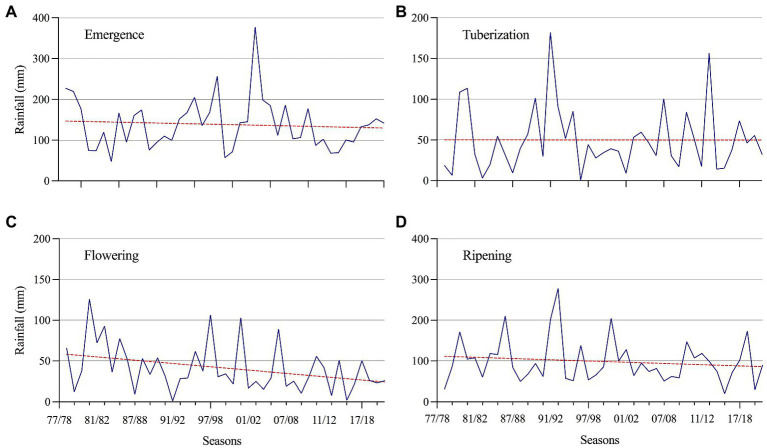
Long-term accumulated rainfall amount in different growth stages of potato: **(A)** Emergence, **(B)** Tuberization, **(C)** Flowering, and **(D)** Ripening from seasons 1977/78 to 2019/20. Each data represent the accumulated rainfall amount during the phenologic stage.

**Figure 3:**
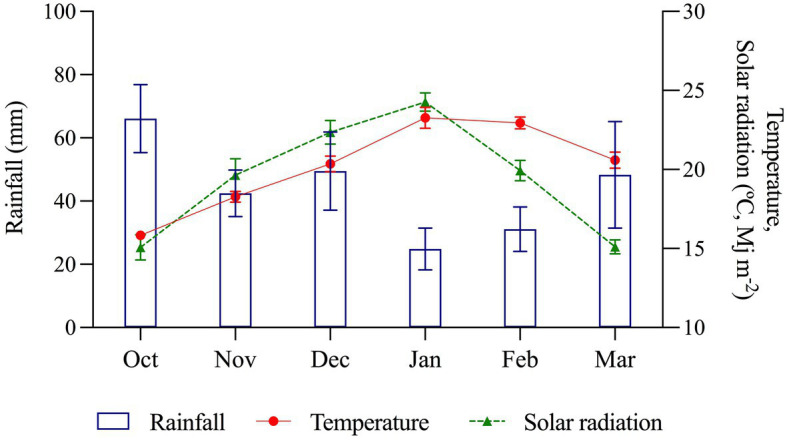
Mean monthly rainfall, temperature, and solar radiation distribution during development stage in potato (2012–13 to 2019–20). Data were provided by the Agrometeorology Network at INIA Remehue. Error bars indicate SE.

The physical properties of volcanic soils in the study area showed low bulk density (0.75 ± 0.04 Mg ha^−1^) and high porosity (67% ± 1.5) in the soil profile (Table 2). The study found decreasing trend of accumulated rainfall during the potato growing season across the last 42 years (Figure 1). The less frequency of accumulated rainfall higher than 400 mm during the growing season is associated with accumulated rainfall lower than 300 mm. Moreover, the average rainfall in the last 4 decades has decreased around 25% in the last 10 years. Figure 2 showed that this decreasing rainfall was more intense during the flowering stage. During the experiment (2012–13 to 2019–20), the accumulated rainfall during the growing season was 242.4 mm (Figure 3). The ambient temperature was 20.5°C, and the mean daily solar radiation was 19.8 Mj m^−2^. Around 42% of rainfall took place in the first 2 months after the planting date. Around 24% (58 mm) occurred between January and February, the tuber-filling and maturation period (flowering to ripening stage) when the average temperature was 23.1°C. Radiation was 22.1 Mj m^−2^ (Figure 3).

**Table 2:** Physical properties of soil profile of the field experiment.

Depth	Bulk density	Porosity	Volumetric SWC	Soil color	Color
cm	Mg m^−3^	%	%	ID	
0–10	0.78 ± 0.01	68 ± 0.34	8.9 ± 1.37	7.5YR 4/4	Brown
10–20	0.78 ± 0.01	68 ± 0.28	14.0 ± 0.71	7.5YR 3/4	Dark yellowish brown
20–30	0.81 ± 0.00	64 ± 0.19	13.6 ± 0.02	7.5YR 4/4	Dark yellowish brown
30–40	0.79 ± 0.00	65 ± 0.14	12.8 ± 1.33	7.5YR 4/4	Dark yellowish brown
40–50	0.76 ± 0.01	66 ± 0.62	15.5 ± 0.65	7.5YR 3/4	Dark yellowish brown
50–60	0.74 ± 0.00	66 ± 0.15	14.7 ± 0.88	7.5YR 3/4	Dark yellowish brown
60–70	0.71 ± 0.01	68 ± 0.26	15.8 ± 0.85	7.5YR 4/4	Dark yellowish brown
70–80	0.71 ± 0.00	68 ± 0.21	16.5 ± 1.52	7.5YR 4/4	Dark brown
80–90	0.69 ± 0.00	69 ± 0.10	19.7 ± 1.36	7.5YR 3/3	Dark brown
90–100	0.72 ± 0.01	67 ± 0.30	24.2 ± 0.47	7.5YR 3/4	Dark brown

SWC, soil water content.

**Figure 4:**
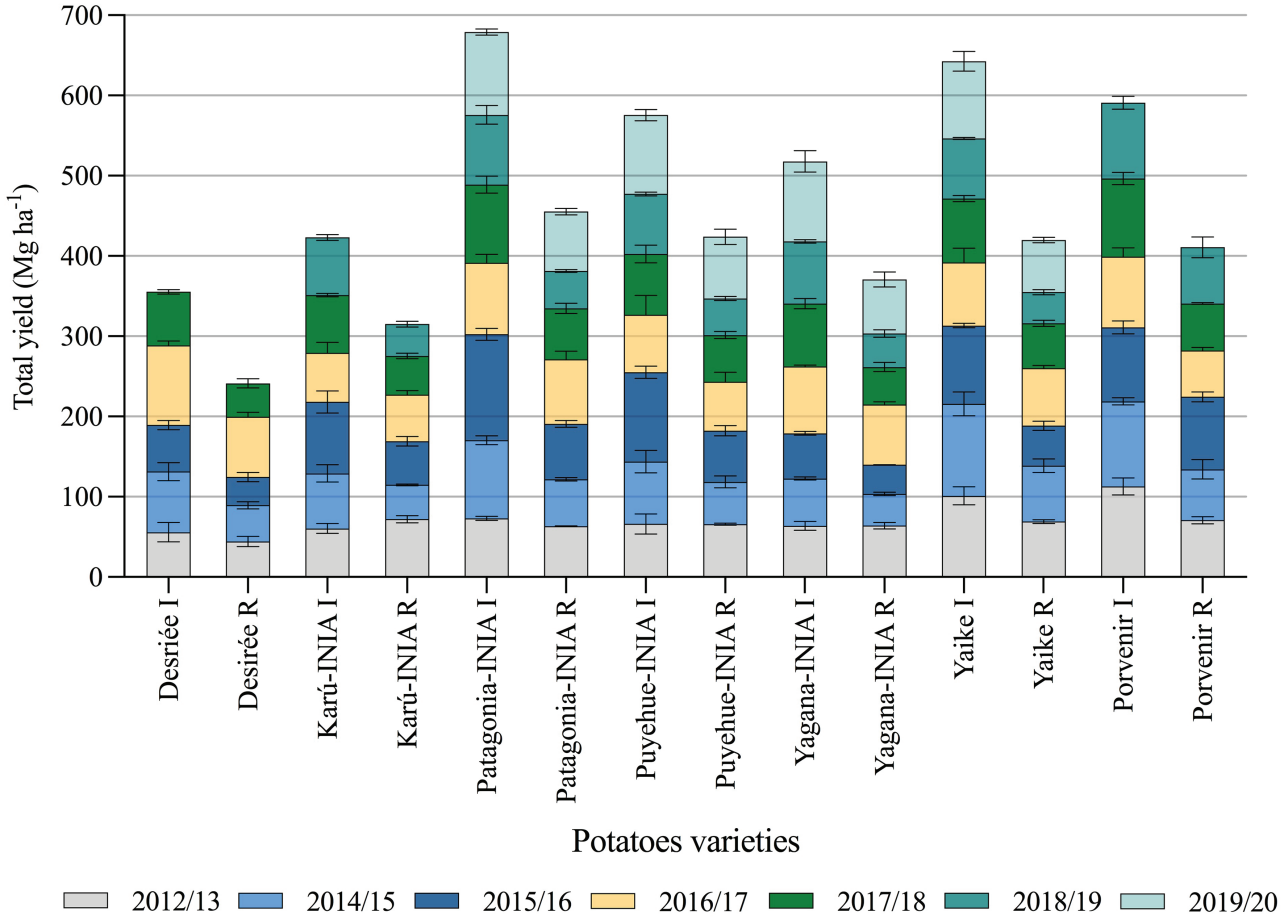
Temporal tuber yield of seven potato varieties under irrigation and rainfed conditions from 2012–13 to 2018–19 season. Desiree and Porvenir with five and six seasons, respectively. Error bars indicate SE. I, irrigation condition; R, rainfed condition.

The average yield of the seven varieties during this period under I conditions was 84 Mg ha^−1^, while the average yield under R conditions was 58 Mg ha^−1^ (Figure 4). The varieties with higher productive potential under I conditions were Porvenir, Patagonia-INIA, Yaike, and Puyehue-INIA, with average yields of 100, 97, 89, and 84 Mg ha^−1^, respectively; in contrast, the varieties Karú-INIA, Yagana-INIA, and Desiree had less productive potential with average yields of 71, 73, and 69 Mg ha^−1^, respectively. Under R conditions, the varieties with more productive potential were Porvenir, Patagonia-INIA, Puyehue-INIA, and Yaike, with average yields of 69, 65, 60, and 59 Mg ha^−1^, respectively. In contrast, the varieties with less productive potential were Karú-INIA, Yagana-INIA, and Desiree, with average yields of 52, 52, and 47 Mg ha^−1^, respectively.

**Figure 5:**
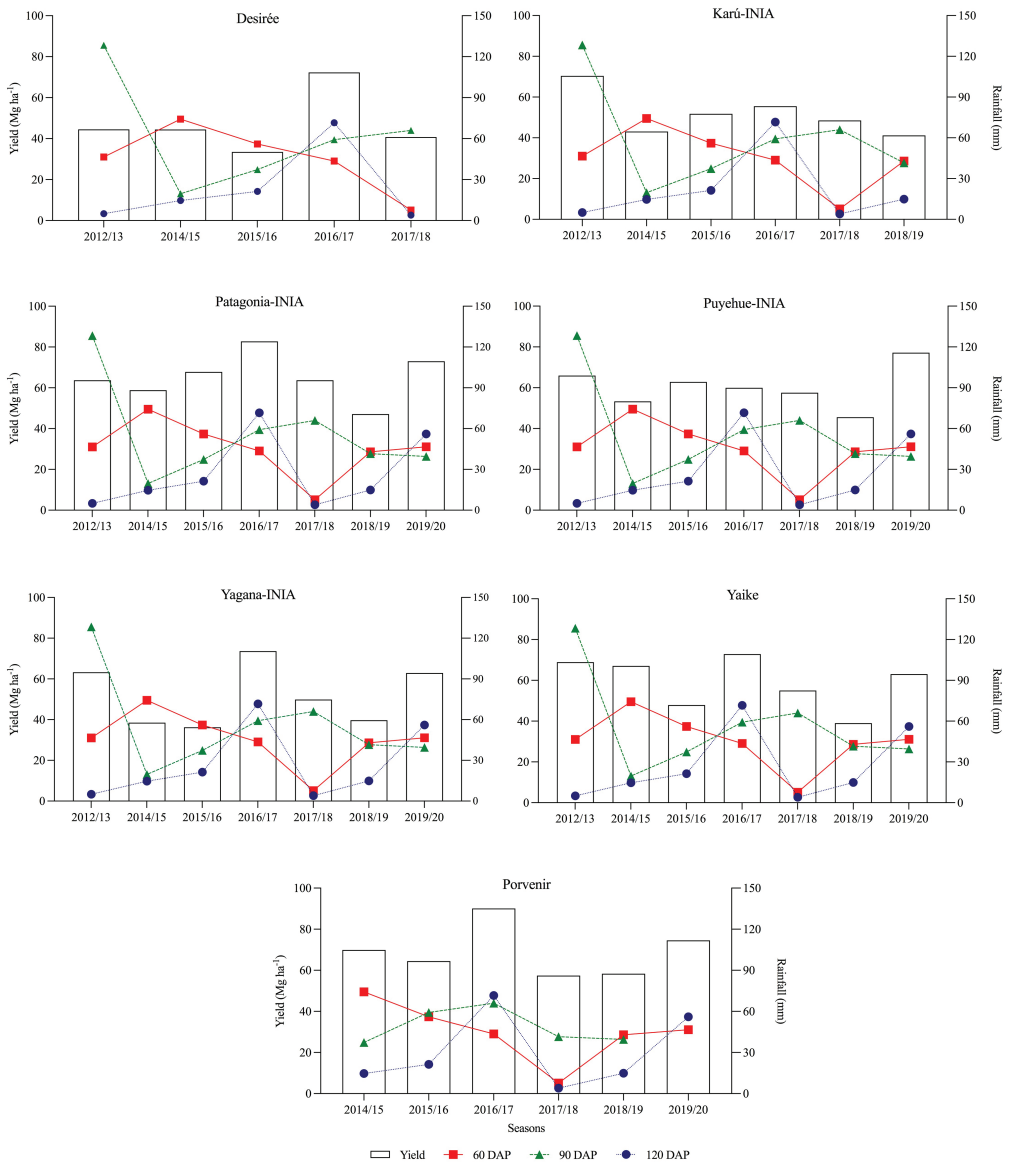
Temporal relationship between potato tuber yield under rainfed conditions and rainfall at 60, 90, and 120 days after planting (DAP), from the 2012–13 to 2019–20 season. 60 DAP: 0–60 days, 90 DAP:60–90 days, and 120 DAP: 90–120 days.

**Figure 6:**
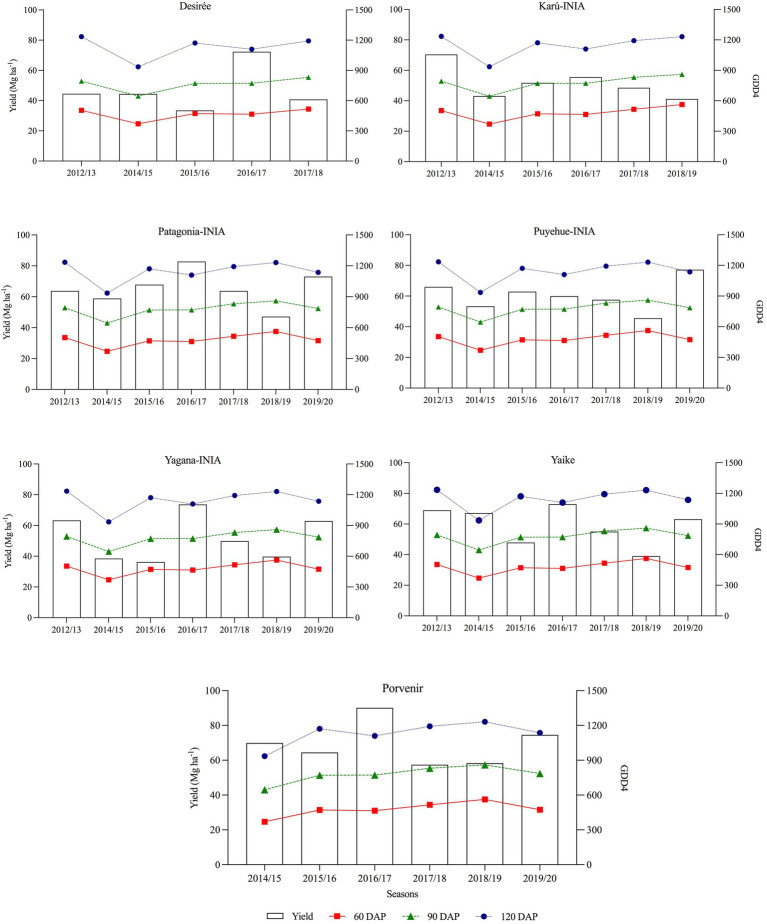
Temporal relationship between potato tuber yield and GDD4 at 60, 90, and 120 DAP, from the 2012–13 to 2019–20 season under rainfed conditions. 60 DAP: 0–60 days, 90 DAP:60–90 days, and 120 DAP: 90–120 days.

**Figure 7:**
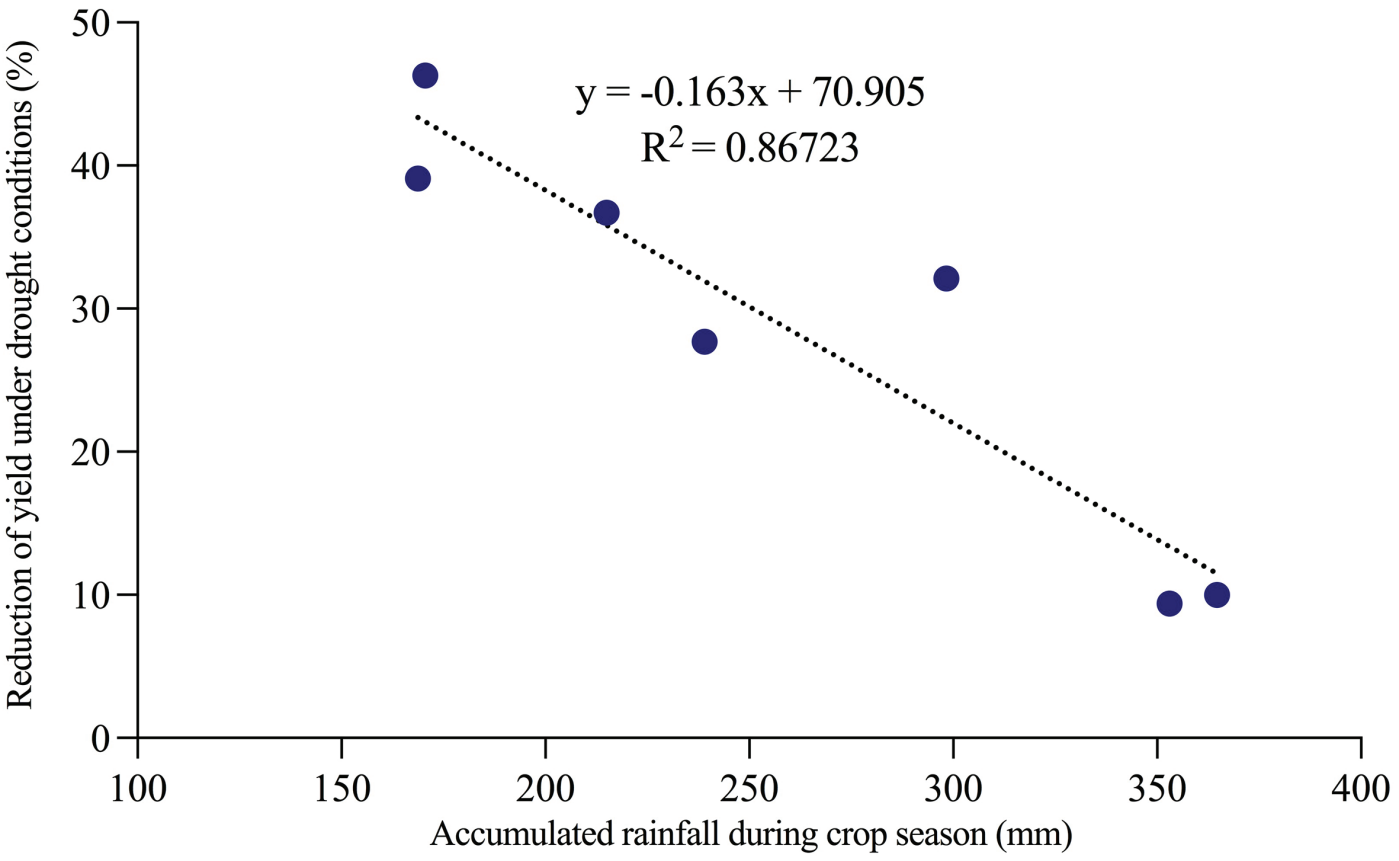
Percentage of reduction of the yield under rainfed conditions compared to irrigated plots concerning the accumulated precipitation during the growing season between 2012–12 and 2019–20 seasons.

Figure 5 shows the temporal relationship between tuber yield under R conditions and rainfall at 0–60, 60–90, and 90–120 DAP. As can be seen, tuber yield increased in years with higher rainfall in the period 90–120 DAP (±60 mm), as occurred in years 2016 (season 2016–17) and 2019 (season 2019–20). GDD4 was similar from one season to another (Figure 6), except the 2014–15 season, which registered the lowest values during the period 90–120 DAP (935) and was also the year with the low tuber yield and precipitation during the same period (Figure 5). Figure 7 shows that when the accumulated rainfall during the crop development exceeded 350 mm, the reduction of tuber yield under rainfed conditions is by 10%. However, when the accumulated rainfall was less than 200 mm, tuber yield decreased by more than 35%.

### Stress Indices and Their Correlations With Tuber Yield

**Figure 8:**
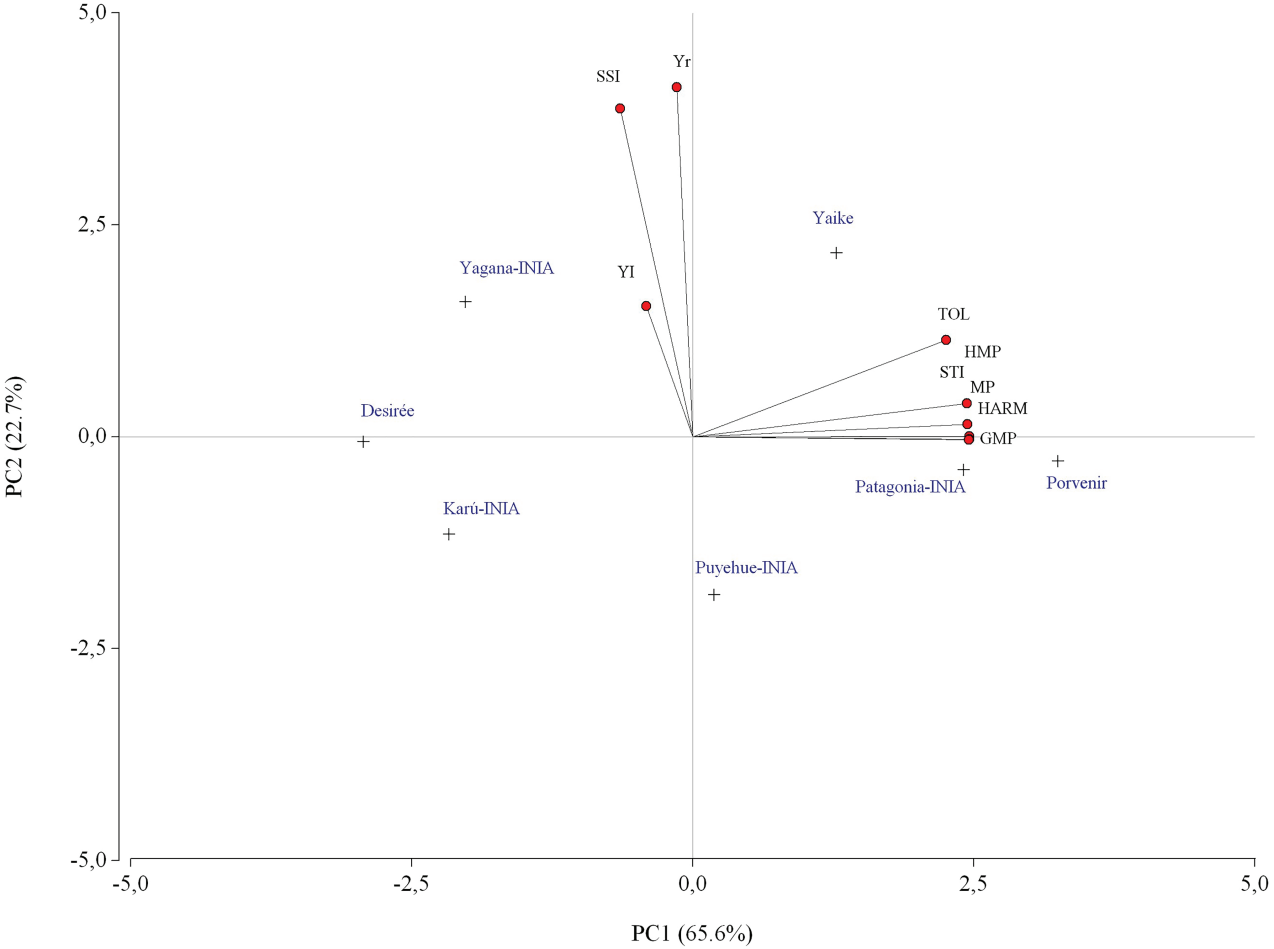
Biplot of the potato varieties under study over different stress tolerance indices under irrigation and rainfed conditions between the 2012–2013 and 2019–2020 seasons.

A PCA was applied to obtain a visual representation of the correlation between the yields of the varieties and the stress tolerance indices. The biplot analysis shown in Figure 8 indicates that PC1 and PC2 explain 88.3% of the total variation and that most of this percentage is accounted for by PC1 (65.6%). There was a strong positive correlation between TOL, MP, GMP, Harm, STI, and HMPF (*r* = >0.92), indicating that these indices are very similar for drought selection. Besides, a weak correlation between YI, Yr, and SSI and the stress indices mentioned before (Table 3). The biplot indicates the association between the stress tolerance indices and varieties in productivity and drought tolerance. The varieties Porvenir, Patagonia-INIA, Yaike, and Puyehue-INIA, indicate a close positive relationship between TOL, MP, GMP, HARM, STI, and HMP as most productive and tolerant to drought conditions. In contrast, Desiree, Yagana-INIA, and Karú-INIA are on the opposite end of the PCA, which indicates that these varieties have negative effects on the dependence of the selection indices, which indicates low productivity and more sensitivity to drought conditions. No associations were found between the indices Yr, SSI, and YI and any of the varieties used in this study, which suggests that these indices are not useful for selecting genotypes or varieties studied under contrasting environmental conditions.

**Table 3:** The correlation coefficient between drought stress conditions and drought tolerance indices between 2012–13 and 2019–20.

	YI	TOL	MP	GMP	HARM	STI	HMP	Yr	SSI
YI	1.00								
TOL	0.04	1.00							
MP	−0.17	0.91	1.00						
GMP	−0.18	0.90	1.00	1.00					
HARM	−0.19	0.90	1.00	1.00	1.00				
STI	−0.09	0.92	0.99	0.99	0.99	1.00			
HMP	−0.17	0.88	0.99	1.00	1.00	0.99	1.00		
Yr	0.24	0.21	−0.06	−0.06	−0.06	0.03	−0.02	1.00	
SSI	0.16	−0.04	−0.26	−0.26	−0.26	−0.18	−0.21	0.95	1.00

YI, yield index; TOL, tolerance index; MP, mean productivity; GMP, geometric mean productivity; HARM, harmonic mean; STI, stress tolerance index; HMP, harmonic mean productivity; Yr, yield reduction; and SSI, stress susceptibility index.

**Figure 9:**
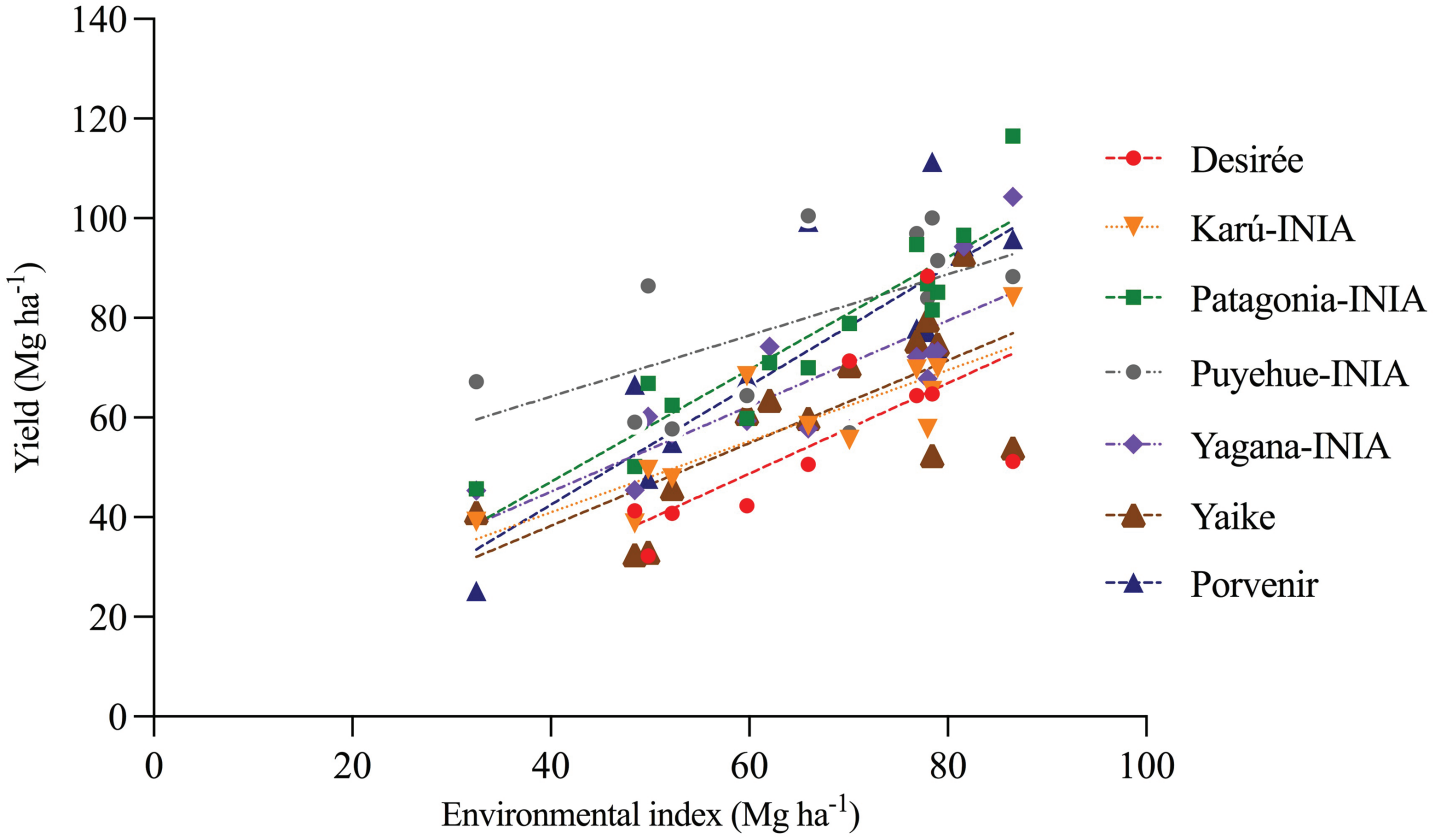
Relationship between yield and environmental index for seven potato varieties.

In Figure 9, the graphs of the average yields of the different varieties in the function of the environmental index (the average yield of all varieties in each environment, considering as an environment the combination of the year and the irrigation or rainfed condition, Table 4) show that the varieties Porvenir and Patagonia-INIA have the highest adjusted regression curves, which indicates that these varieties are more tolerant to water deficits and had higher yields over the years of the study and the distinct climatic conditions. This, in turn, indicates that these varieties have higher stress tolerance indices and a good response to improved conditions with irrigation. Coincidentally, the yield regression curve in the context of the environmental indices of varieties Desiree and Karú-INIA, which were less correlated with the drought tolerance index and showed had the lowest curves (Figure 9).

**Table 4:** Environmental index (average yield of seven varieties) per year for irrigated and rainfed conditions.

Year	Season pp	E. Index (Mg ha^−1^)	
		Rainfed	Irrigated
2012	353	59.7	65.9
2014	215	49.6	78.4
2015	169	52.7	86.5
2016	365	70.1	77.9
2017	298	52.2	76.9
2018	171	42.3	78.9
2019	239	67.9	93.9
Average	258.6	56.4	79.8

The regression between the environmental index and the number of tubers did not result in a significant relationship, indicating that this yield component was not affected by the effects of the irrigated and rainfed conditions in this agroclimatic context (Table 5). In contrast, there was a significant relationship between the average weight of the tubers of all the varieties and the environmental index (*p* < 0.0001), with determination coefficients similar to those between the environmental index and yield, indicating that the tuber weight component was affected by differences in water availability.

**Table 5:** Coefficients of the regression between yield and environmental index for seven potato varieties.

Cultivar	Average yield^*^	Regression parameters in function of the environmental index (E. index)								
		yield vs. E. index			number of tubers vs. E. index			weight of individual tuber vs. E. index		
		*R*^2^	*b* (slope)	*p*	*R*^2^	*b* (slope)	*p*	*R*^2^	*b* (slope)	*p*
Desiree	54.7 ± 17.5	0.46	0.91	<0.0001	0.28	NC	0.0026	0.5	0.73	<0.0001
Karú-INIA	58.7 ± 14.4	0.58	0.69	<0.0001	0.16	NC	>0.05	0.49	0.58	<0.0001
Patagonia-INIA	76.2 ± 19.9	0.75	1.13	<0.0001	0.05	NC	>0.05	0.86	0.76	<0.0001
Puyehue-INIA	67.25 ± 18.5	0.51	0.86	<0.0001	0.05	NC	>0.05	0.61	0.54	<0.0001
Yagana-INIA	59.69 ± 18.1	0.49	0.83	<0.0001	0.09	NC	>0.05	0.59	0.51	<0.0001
Yaike	73.28 ± 23.4	0.61	1.19	<0.0001	0.05	NC	>0.05	0.81	0.59	<0.0001
Porvenir	79.42 ± 17.9	0.74	0.94	<0.0001	0.06	NC	>0.05	0.67	0.72	<0.0001

* Average yield for all years and treatments.

### Drought Effects on the Tuber Size Distribution

**Figure 10:**
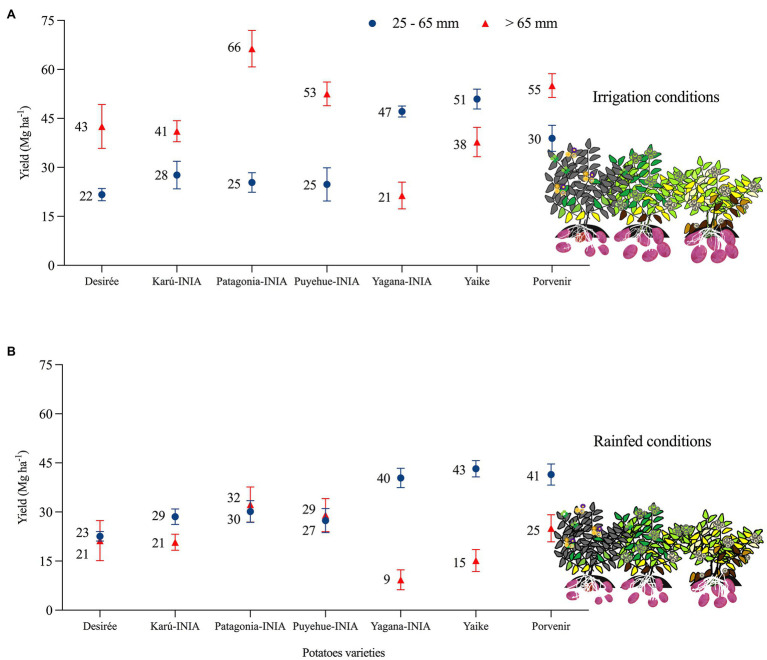
Average tuber size distribution of seven potato varieties under irrigation and rainfed conditions from 2012–14 to 2019–20. (A) Yield of marketable tubers: > 65 mm. (B) Yield of seed tubers (25–65 mm). Error bars indicate SE (*p* < 0.05).

The previous results showed that yield was reduced under water limiting conditions depending on the drought severity. However, tuber size was the most affected under water limiting conditions in the different varieties. Figure 10 shows the average yield for the tuber size distribution of seed (25–65 mm) and marketable tubers (>65 mm) over the years of the study under both conditions (irrigation and rainfed). Drought stress significantly reduced the marketable tubers (>65 mm) by 50–60%. However, under irrigation, not all the varieties were marketable, like Yagana-INIA and Yaike, which are more productive for tuber seed production. The results also indicated that with irrigation, the productivity of Patagonia-INIA and Porvenir differs depending on the tuber size distribution. At the same time, Patagonia-INIA has a higher marketable production by 73%, for Porvenir was by 59%.

## Discussion

### Tuber Yield Productivity Under Irrigation vs. Rainfed Conditions

The results of the medium-term trial showed the responses of the seven high-yield varieties to water deficit (Figure 4), indicating that some varieties are more tolerant to water stress than others. Therefore, variability for drought tolerance is affected differently between cultivars (Aliche et al., 2019). However, it was observed that irrigation can increase yield in these soils by 30% (Figure 4). Although Andisols are characterized by the capacity to store and transport water, air, and nutrients, which maximizes plant yield (Valle et al., 2018), the physical properties of the soil capacity (air and water) are sensitive to management practices (Ordoñez et al., 2018), which the limits of storage and transport of water will depend on the pore system (Clunes et al., 2021). Water availability is limiting in the potato growing season in this region, despite the ability of soils to store moisture. Therefore, rainfall during the growing season is pivotal for yield, even in these cool temperate areas with favorable soils for potatoes (Liang et al., 2020). In addition, extremely high soil temperatures and low water content disfavor tuber formation (Liang et al., 2020). Even short periods of water stress can significantly reduce tuber yield, as was observed in our study (Zhang et al., 2018). Comparing yields under irrigation vs. rainfed conditions over seven seasons, the varieties Patagonia-INIA, Puyehue-INIA, Yagana-INIA, and Yaike registered differences in yields of 220, 168, 149, and 210 Mg ha^−1^, respectively. Over five and six seasons, the varieties Desiree and Karú-INIA registered differences in yields of 110 and 118 Mg ha^−1^, respectively, while the last variety to be released to the market, Porvenir, registered a difference of 186 Mg ha^−1^ over six seasons. This not only demonstrates that water deficits significantly affect tuber growth and development (Karam et al., 2014; Ambrosone et al., 2016) but can also cause the loss of yields for one or two seasons over the medium-term. This was observed with the yields of Patagonia-INIA, Yaike, and Puyehue-INIA, which registered accumulated yields of 677, 624, and 591 Mg ha^−1^, respectively under irrigation. In comparison, Patagonia-INIA reached 601 Mg ha^−1^ over six seasons (Figure 4). Figure 3 shows that the lowest precipitation levels occurred in January and February, which typically follow flowering in this region. By this time, the number of tubers has already been fixed. Still, during this period, tubers acquire their final weight, which concurs with the environmental index and explains the differences in yields under irrigation and rainfed conditions. The results in a yield of high productive varieties by 7 years indicate an important limiting for yield in no irrigated crops, even considering that south of Chile is an area with big yield potential for potatoes where this species is typically grown in drylands. Data of accumulated rainfall during the potato growing season during the last 40 years (Figures 1, 2) show a tendency to lower amounts from past to present. Therefore, potato improvement for drought tolerance and implementation of irrigation systems in this crop is essential to obtain marketable yields and avoid the risk of failure of the crop in years with low precipitation. The situation will probably be more pronounced in the future, given the trends in the last decades (Raymundo et al., 2014; Aliche et al., 2019; Hill et al., 2021). These results exemplify how much we can lose in productivity in dryland conditions even in one of the most favorable areas for growing potatoes globally and how risky this situation can be for the performance of the potato farms in the future.

### Effects of Drought Conditions on Tuber Yield

Drought conditions lead to a reduction in total tuber yield and marketable yield (Figure 10). The yields of the potato varieties increased under conditions of water stress when there was precipitation exceeded 60 mm in the first 90–120 DAP (Figure 5), which indicates that the important phenological stages and types of varieties should be considered before applying deficit irrigation to maximize productivity (Martínez-Romero et al., 2019). Karam et al. (2014) observed that water deficits during tuber development in semiarid climates can reduce yields by 12%, while water deficits during tuber filling decreased yields by 42%. As observed in Figure 10, the effect of drought during the tuber-filling period reduces tuber size distribution and tuber yield (Aliche et al., 2018). Yagana-INIA and Yaike were more productive for tuber seed production across the years of study. Aliche et al. (2019) indicate that cultivars that make more tubers than they can bulk during the growing season would distribute smaller sizes, especially under rainfed conditions.

The highest yields under the I conditions compared to the R conditions occurred in the 2014–15 season (Figure 4) and coincided with the lowest rainfall and GDD4 at 90 DAP, and 120 DAP (Figures 5, 6) occurred during the tuber-filling period. This could also be associated with the warmest temperature (23°C) and highest levels of solar radiation, 24.3 (Mj m^−2^), in January (Figure 3). Van Harsselaar et al. (2021) mentioned that tuber growth is inhibited under combined drought and heat stress, with elevated temperatures of 30°C during the day. Timlin et al. (2006) indicated that the supply of photosynthates to tuber decreases with higher temperatures since the optimum temperature for biomass accumulation in potato is 20°C (Monneveux et al., 2013; Liang et al., 2020). Moreover, drought on tuberization reduces tuber formation and has a strong effect on tuber yield (Aliche et al., 2018). However, although the climatic conditions, especially precipitation, varied during the phenological period of the crop and during the years of the study, the accumulations of GDD4 were similar in the study area (Figure 6). Similar values were observed with the variety Desiree in a Mediterranean climate, indicating a weak correlation between yield and exposure of the plant to GDD (Danieli et al., 2018). Finally, the stress tolerance indices confirmed which varieties are best and least adapted to water deficit. The variables which contributed more positively to PC1 were GMP, HARM, MP, HMP, and TOL, which indicated an association between these indices and the general adaptability of the varieties to stressful environments. The varieties Porvenir, Patagonia-INIA, and Yaike, were the most adaptable (Figure 8; Table 3) and had the best drought tolerance indices. This confirms that these indices identify genotypes with high yield potential and tolerance to water stress. The GMP was the best-adapted index to assess relative yield (Mohammed and Kadhem, 2017). SSI, Yr, and YI are probably only useful to identify genotypes that behave well under stressful conditions but are not appropriate to identify genotypes that respond to improvements in environmental conditions. Krishnamurthy et al. (2016) indicated that SSI often fails to identify genotypes with high yields and stress tolerance potentials. Thus, Nikneshan et al. (2019) have also indicated that SSI, Yr, and YI work best when the changes in the yield of the genotype are lower and more stable over time.

## Conclusion

The results in terms of yield-high varieties by 7 years indicate an important limiting in non-irrigated tuber yield, which decreased by 27 and 34% under rainfed conditions. However, tuber size distribution was the most affected under water limiting conditions, significantly reducing the marketable tubers (>65 mm) by 50–60%. The varieties Porvenir, Patagonia-INIA, and Yaike, had higher yields under conditions of irrigation and rainfed. However, it was observed that the varieties had high yields under water stress conditions when precipitation of more than 60 mm occurred between 60 and 120 DAP. The best indices to study drought tolerance were TOL, MP, GMP, Harm, STI, and HMP. Our study suggests that even in cool temperate areas with favorable soil properties for potatoes, as Andisols, low rainfall can produce a serious constraint for productivity. Therefore, combining varieties with more water stress tolerance and providing supplementary irrigation during the dry period can optimize the yield under rainfed conditions.

## Data Availability Statement

The original contributions presented in the study are included in the article/supplementary material, further inquiries can be directed to the corresponding author.

## Author Contributions

IM, MM, and MU contributed to conception and design of the study and organized the database. IM, MM, and IA performed the statistical analysis and wrote sections of the manuscript. IM wrote the first draft of the manuscript. All authors contributed to the article and approved the submitted version.

### Conflict of Interest

The authors declare that the research was conducted in the absence of any commercial or financial relationships that could be construed as a potential conflict of interest.
